# Genetic Detection and Characterization of Lujo Virus, a New Hemorrhagic Fever–Associated Arenavirus from Southern Africa

**DOI:** 10.1371/journal.ppat.1000455

**Published:** 2009-05-29

**Authors:** Thomas Briese, Janusz T. Paweska, Laura K. McMullan, Stephen K. Hutchison, Craig Street, Gustavo Palacios, Marina L. Khristova, Jacqueline Weyer, Robert Swanepoel, Michael Egholm, Stuart T. Nichol, W. Ian Lipkin

**Affiliations:** 1 Center for Infection and Immunity, Mailman School of Public Health, Columbia University, New York, New York, United States of America; 2 Special Pathogens Unit, National Institute for Communicable Diseases of the National Health Laboratory Service, Sandringham, South Africa; 3 Special Pathogens Branch, Division of Viral and Rickettsial Diseases, Centers for Disease Control and Prevention, Atlanta, Georgia, United States of America; 4 454 Life Sciences, Branford, Connecticut, United States of America; 5 Biotechnology Core Facility Branch, Centers for Disease Control and Prevention, Atlanta, Georgia, United States of America; University of California Irvine, United States of America

## Abstract

Lujo virus (LUJV), a new member of the family *Arenaviridae* and the first hemorrhagic fever–associated arenavirus from the Old World discovered in three decades, was isolated in South Africa during an outbreak of human disease characterized by nosocomial transmission and an unprecedented high case fatality rate of 80% (4/5 cases). Unbiased pyrosequencing of RNA extracts from serum and tissues of outbreak victims enabled identification and detailed phylogenetic characterization within 72 hours of sample receipt. Full genome analyses of LUJV showed it to be unique and branching off the ancestral node of the Old World arenaviruses. The virus G1 glycoprotein sequence was highly diverse and almost equidistant from that of other Old World and New World arenaviruses, consistent with a potential distinctive receptor tropism. LUJV is a novel, genetically distinct, highly pathogenic arenavirus.

## Author Summary

In September and October 2008, five cases of undiagnosed hemorrhagic fever, four of them fatal, were recognized in South Africa after air transfer of a critically ill index case from Zambia. Serum and tissue samples from victims were subjected to unbiased pyrosequencing, yielding within 72 hours of sample receipt, multiple discrete sequence fragments that represented approximately 50% of a prototypic arenavirus genome. Thereafter, full genome sequence was generated by PCR amplification of intervening fragments using specific primers complementary to sequence obtained by pyrosequencing and a universal primer targeting the conserved arenaviral termini. Phylogenetic analyses confirmed the presence of a new member of the family *Arenaviridae*, provisionally named Lujo virus (LUJV) in recognition of its geographic origin (Lusaka, Zambia, and Johannesburg, South Africa). Our findings enable the development of specific reagents to further investigate the reservoir, geographic distribution, and unusual pathogenicity of LUJV, and confirm the utility of unbiased high throughput pyrosequencing for pathogen discovery and public health.

## Introduction

Members of the genus *Arenavirus*, comprising currently 22 recognized species (http://www.ictvonline.org/virusTaxonomy.asp?version=2008), are divided into two complexes based on serologic, genetic, and geographic relationships [1],[2]: the New World (NW) or Tacaribe complex, and the Old World (OW) or Lassa-Lymphocytic choriomeningitis complex that includes the ubiquitous arenavirus type-species *Lymphocytic choriomeningitis virus* (LCMV; [3]). The RNA genome of arenaviruses is bi-segmented, comprising a large (L) and a small (S) segment that each codes for two proteins in ambisense coding strategy [4],[5]. Despite this coding strategy, the *Arenaviridae* are classified together with the families *Orthomyxoviridae* and *Bunyaviridae* as segmented single-strand, negative sense RNA viruses.

The South American hemorrhagic fever viruses Junin (JUNV; [6],[7]), Machupo (MACV; [8]), Guanarito (GTOV; [9]) and Sabia virus (SABV, [10]), and the African Lassa virus (LASV [11]), are restricted to biosafety level 4 (BSL-4) containment due to their associated aerosol infectivity and rapid onset of severe disease. With the possible exception of NW Tacaribe virus (TCRV; [12]), which has been isolated from bats (*Artibeus* spp.), individual arenavirus species are commonly transmitted by specific rodent species wherein the capacity for persistent infection without overt disease suggests long evolutionary adaptation between the agent and its host [1], [13]–[16]. Whereas NW arenaviruses are associated with rodents in the *Sigmodontinae* subfamily of the family *Cricetidae*, OW arenaviruses are associated with rodents in the *Murinae* subfamily of the family *Muridae*.

**Figure 1:**
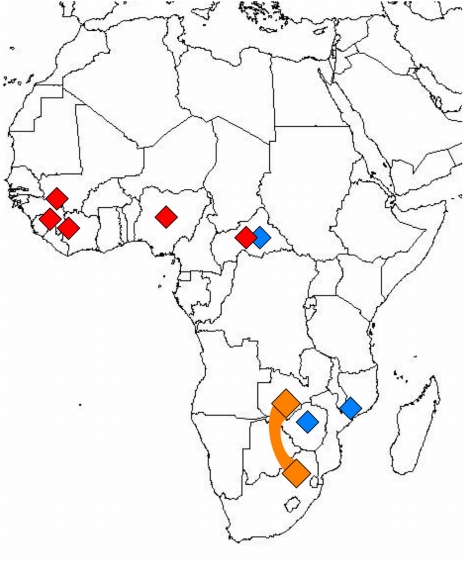
Geographic distribution of African arenaviruses. MOBV, MOPV, and IPPYV (blue) have not been implicated in human disease; LASV (red) can cause hemorrhagic fever. The origin of the LUJV index and secondary and tertiary cases linked in the 2008 outbreak are indicated in gold.

Humans are most frequently infected through contact with infected rodent excreta, commonly via inhalation of dust or aerosolized virus-containing materials, or ingestion of contaminated foods [13]; however, transmission may also occur by inoculation with infected body fluids and tissue transplantation [17]–[19]. LCMV, which is spread by the ubiquitous *Mus musculus* as host species and hence found world-wide, causes symptoms in humans that range from asymptomatic infection or mild febrile illness to meningitis and encephalitis [13]. LCMV infection is only rarely fatal in immunocompetent adults; however, infection during pregnancy bears serious risks for mother and child and frequently results in congenital abnormalities. The African LASV, which has its reservoir in rodent species of the *Mastomys* genus, causes an estimated 100,000–500,000 human infections per year in West African countries (Figure 1). Although Lassa fever is typically sub-clinical or associated with mild febrile illness, up to 20% of cases may have severe systemic disease culminating in fatal outcome [20],[21]. Three other African arenaviruses are not known to cause human disease: Ippy virus (IPPYV; [22],[23]), isolated from *Arvicanthis* spp. and Mobala virus (MOBV; [24]) isolated from *Praomys* spp. in the Central African Republic (CAR); and Mopeia virus (MOPV) that like LASV is associated with members of the genus *Mastomys*, and was reported from Mozambique [25] and Zimbabwe [26], although antibody studies suggest that MOPV and LASV may also circulate in CAR [27] where the geographies of these viruses appear to overlap (Figure 1). Up to present, there have been no published reports of severe human disease associated with arenaviruses isolated from southern Africa.

In September 2008 an outbreak of unexplained hemorrhagic fever was reported in South Africa [28]. The index patient was airlifted in critical condition from Zambia on September 12 to a clinic in Sandton, South Africa, after infection from an unidentified source. Secondary infections were recognized in a paramedic (case 2) who attended the index case during air transfer from Zambia, in a nurse (case 3) who attended the index case in the intensive care unit in South Africa, and in a member of the hospital staff (case 4) who cleaned the room after the index case died on September 14. One case of tertiary infection was recorded in a nurse (case 5) who attended case 2 after his transfer from Zambia to Sandton on September 26, one day before barrier nursing was implemented. The course of disease in cases 1 through 4 was fatal; case 5 received ribavirin treatment and recovered. A detailed description of clinical and epidemiologic data, as well as immunohistological and PCR analyses that indicated the presence of an arenavirus, are reported in a parallel communication (Paweska et al., Emerg. Inf. Dis., submitted). Here we report detailed genetic analysis of this novel arenavirus.

## Results/Discussion

### Rapid identification of a novel pathogen through unbiased pyrosequencing

**Figure 2:**
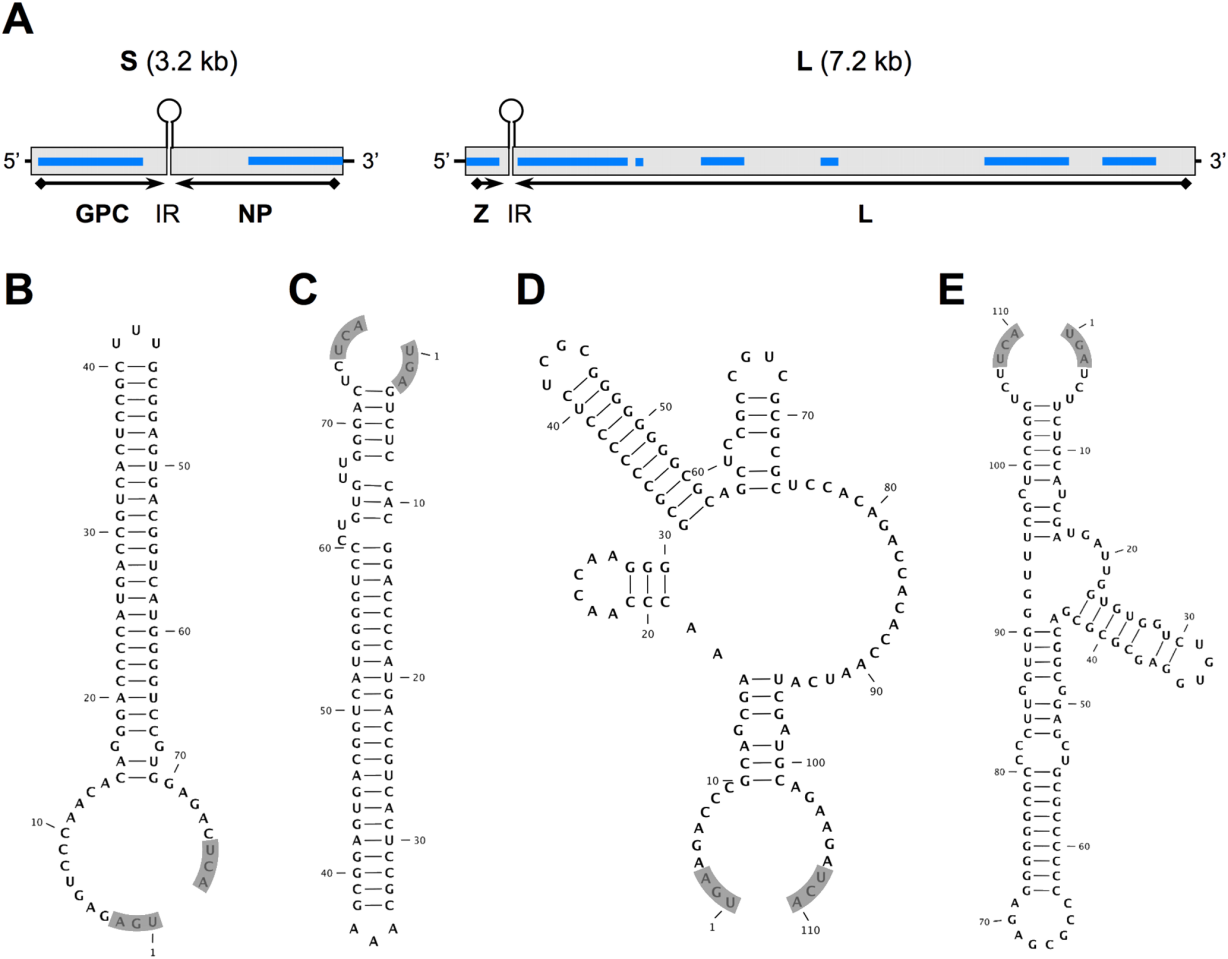
LUJV genome organization and potential secondary structure of intergenic regions. Open reading frames (ORF) for the glycoprotein precursor GPC, the nucleoprotein NP, the matrix protein analog Z, and the polymerase L, and their orientation are indicated (A); blue bars represent sequences obtained by pyrosequencing from clinical samples. Secondary structure predictions of intergenic regions (IR) for S (B, C) and L segment sequence (D, E) in genomic (B, D) and antigenomic orientation (C, E) were analyzed by mfold; shading indicates the respective termination codon (opal, position 1), and its reverse-complement, respectively.

RNA extracts from two post-mortem liver biopsies (cases 2 and 3) and one serum sample (case 2) were independently submitted for unbiased high-throughput pyrosequencing. The libraries yielded between 87,500 and 106,500 sequence reads. Alignment of unique singleton and assembled contiguous sequences to the GenBank database (http://www.ncbi.nlm.nih.gov/Genbank) using the Basic Local Alignment Search Tool (blastn and blastx; [29]) indicated coverage of approximately 5.6 kilobases (kb) of sequence distributed along arenavirus genome scaffolds: 2 kb of S segment sequence in two fragments, and 3.6 kb of L segment sequence in 7 fragments (Figure 2). The majority of arenavirus sequences were obtained from serum rather than tissue, potentially reflecting lower levels of competing cellular RNA in random amplification reactions.

### Full genome characterization of a newly identified arenavirus

Sequence gaps between the aligned fragments were rapidly filled by specific PCR amplification with primers designed on the pyrosequence data at both, CU and CDC. Terminal sequences were added by PCR using a universal arenavirus primer, targeting the conserved viral termini (5′-CGC ACM GDG GAT CCT AGG C, modified from [30]) combined with 4 specific primers positioned near the ends of the 2 genome segments. Overlapping primer sets based on the draft genome were synthesized to facilitate sequence validation by conventional dideoxy sequencing. The accumulated data revealed a classical arenavirus genome structure with a bi-segmented genome encoding in an ambisense strategy two open reading frames (ORF) separated by an intergenic stem-loop region on each segment (Figure 2) (GenBank Accession numbers FJ952384 and FJ952385).

Our data represent genome sequences directly obtained from liver biopsy and serum (case 2), and from cell culture isolates obtained from blood at CDC (case 1 and 2), and from liver biopsies at NICD (case 2 and 3). No sequence differences were uncovered between virus detected in primary clinical material and virus isolated in cell culture at the two facilities. In addition, no changes were detected between each of the viruses derived from these first three cases. This lack of sequence variation is consistent with the epidemiologic data, indicating an initial natural exposure of the index case, followed by a chain of nosocomial transmission among subsequent cases.

### Lujo virus (LUJV) is a novel arenavirus

**Figure 3:**
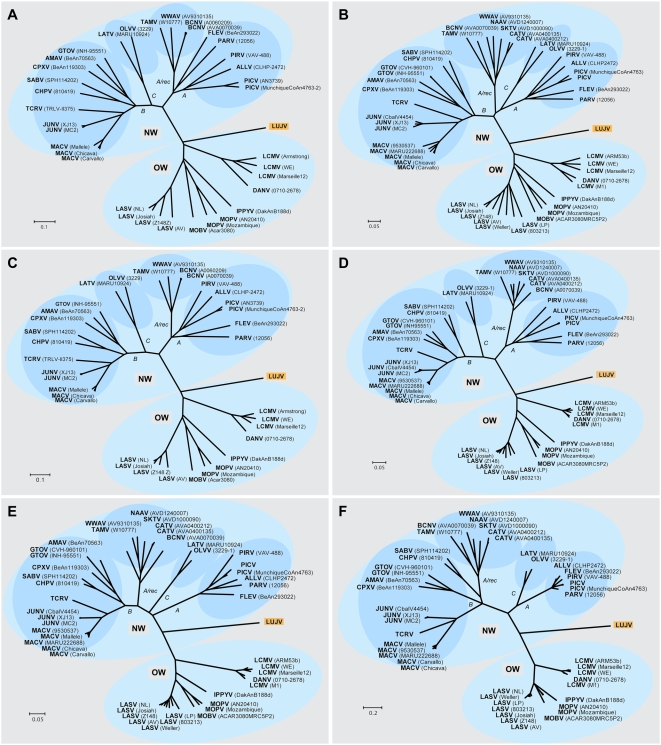
Phylogenetic analyses of LUJV. Phylogenetic relationships of LUJV were inferred based on full L (A) and S segment nucleotide sequence (B), as well as on deduced amino acid sequences of L (C), NP (D), Signal/G2 (E) and G1 (F) ORF's. Phylogenies were reconstructed by neighbor-joining analysis applying a Jukes-Cantor model; the scale bar indicates substitutions per site; robust boostrap support for the positioning of LUJV was obtained in all cases (>98% of 1000 pseudoreplicates). GenBank Accession numbers for reference sequences are: ALLV CLHP2472 (AY216502, AY012687); AMAV BeAn70563 (AF512834); BCNV AVA0070039 (AY924390, AY922491), A0060209 (AY216503); CATV AVA0400135 (DQ865244), AVA0400212 (DQ865245); CHPV 810419 (EU, 260464, EU260463); CPXV BeAn119303 (AY216519, AF512832); DANV 0710-2678 (EU136039, EU136038); FLEV BeAn293022 (EU627611, AF512831); GTOV INH-95551 (AY358024, AF485258), CVH-960101 (AY497548); IPPYV DakAnB188d (DQ328878, DQ328877); JUNV MC2 (AY216507, D10072), XJ13 (AY358022, AY358023), CbalV4454 (DQ272266); LASV LP (AF181853), 803213 (AF181854), Weller (AY628206), AV (AY179171, AF246121), Z148 (AY628204, AY628205), Josiah (U73034, J043204), NL (AY179172, AY179173); LATV MARU10924 (EU627612, AF485259); LCMV Armstrong (AY847351), ARM53b (M20869), WE (AF004519, M22138), Marseille12 (DQ286932, DQ286931), M1 (AB261991); MACV Carvallo (AY619642, AY619643), Chicava (AY624354, AY624355), Mallele (AY619644, AY619645), MARU222688 (AY922407), 9530537 (AY571959); MOBV ACAR3080MRC5P2 (DQ328876, AY342390); MOPV AN20410 (AY772169, AY772170), Mozambique (DQ328875, DQ328874); NAAV AVD1240007 (EU123329); OLVV 3229-1 (AY216514, U34248); PARV 12056 (EU627613, AF485261); PICV (K02734), MunchiqueCoAn4763 (EF529745, EF529744), AN3739 (AF427517); PIRV VAV-488 (AY216505, AF277659); SABV SPH114202 (AY358026, U41071); SKTV AVD1000090 (EU123328); TAMV W10777 (EU627614, AF512828); TCRV (J04340, M20304); WWAV AV9310135 (AY924395, AF228063).

Phylogenetic trees constructed from full L or S segment nucleotide sequence show LUJV branching off the root of the OW arenaviruses, and suggest it represents a highly novel genetic lineage, very distinct from previously characterized virus species and clearly separate from the LCMV lineage (Figure 3A and 3B). No evidence of genome segment reassortment is found, given the identical placement of LUJV relative to the other OW arenaviruses based on S and L segment nucleotide sequences. In addition, phylogenetic analysis of each of the individual ORFs reveals similar phylogenetic tree topologies. A phylogenetic tree constructed from deduced L-polymerase amino acid (aa) sequence also shows LUJV near the root of the OW arenaviruses, distinct from characterized species, and separate from the LCMV branch (Figure 3C). A distant relationship to OW arenaviruses may also be inferred from the analysis of Z protein sequence (Figure S1). The NP gene sequence of LUJV differs from other arenaviruses from 36% (IPPYV) to 43% (TAMV) at the nucleotide level, and from 41% (MOBV/LASV) to 55% (TAMV) at the aa level (Table S1). This degree of divergence is considerably higher than both, proposed cut-off values within (<10–12%), or between (>21.5%) OW arenavirus species [31],[32], and indicates a unique phylogenitic position for LUJV (Figure 3D). Historically, phylogenetic assignments of arenaviruses have been based on portions of the NP gene [1],[33], because this is the region for which most sequences are known. However, as more genomic sequences have become available, analyses of full-length GPC sequence have revealed evidence of possible relationships between OW and NW arenaviruses not revealed by NP sequence alone [34]. Because G1 sequences are difficult to align some have pursued phylogenetic analyses by combining the GPC signal peptide and the G2 sequence for phylogenetic analysis [16]. We included in our analysis the chimeric signal/G2 sequence (Figure 3E) as well as the receptor binding G1 portion (Figure 3F); both analyses highlighted the novelty of LUJV, showing an almost similar distance from OW as from NW viruses.

### Protein motifs potentially relevant to LUJV biology

**Figure 4:**
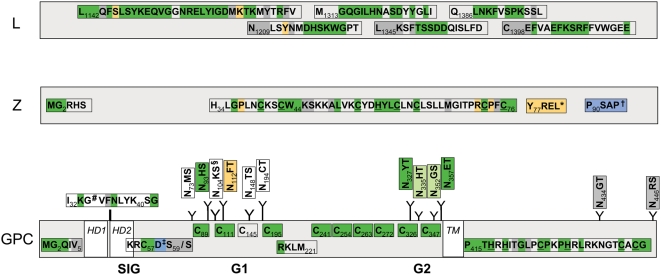
Schematic of conserved protein motifs. Conservation of LUJV amino acid motifs with respect to all other (green highlight), to OW (yellow highlight), or to NW (blue highlight) arenaviruses is indicated; grey highlight indicates features unique to LUJV. Polymerase motifs pre-A (L_1142_), A (N_1209_), B (M_1313_), C (L_1345_), D (Q_1386_), and E (C_1398_) are indicated for the L ORF; potential myristoylation site G_2_, the RING motif H_34_/C_76_, and potential late domains YXXL an PSAP are indicated for the Z ORF; and myristoylation site G_2_, posttranslational processing sites for signalase (S_59_/S_60_) and S1P cleavage (RKLM_221_), CTL epitope (I_32_), zinc finger motif P_415_/G_440_, as well as conserved cysteine residues and glycosylations sites (Y) are indicated for GPC. * late domain absent in NW viruses and DANV; † PSAP or PTAP in NW viruses, except in PIRV and TCRV (OW viruses: PPPY); # G in all viruses except LCMV ( = A); ‡ D in NW clade A only; § conserved with respect to OW, and NW clade A and C; *HD*, hydrophobic domain; *TM*, transmembrane anchor.

Canonical polymerase domains pre-A, A, B, C, D, and E [35]–[37] are well conserved in the L ORF of LUJV (256 kDa, pI = 6.4; Figure 4). The Z ORF (10.5 kDa, pI = 9.3) contains two late domain motifs like LASV; however, in place of the PTAP motif found in LASV, that mediates recognition of the tumor susceptibility gene 101, Tsg101 [38], involved in vacuolar protein sorting [39],[40], LUJV has a unique Y_77_REL motif that matches the YXXL motif of the retrovirus equine infectious anemia virus [41], which interacts with the clathrin adaptor protein 2 (AP2) complex [42]. A Tsg101-interacting motif, P_90_SAP, is found in LUJV in position of the second late domain of LASV, PPPY, which acts as a Nedd4-like ubiquitin ligase recognition motif [43]. The RING motif, containing conserved residue W_44_ [44], and the conserved myristoylation site G_2_ are present [45]–[47] (Figure 4). The NP of LUJV (63.1 kDa, pI = 9.0) contains described aa motifs that resemble mostly OW arenaviruses [48], including a cytotoxic T-lymphocyte (CTL) epitope reported in LCMV (GVYMGNL; [49]), corresponding to G_122_VYRGNL in LUJV, and a potential antigenic site reported in the N-terminal portion of LASV NP (RKSKRND; [50]), corresponding to R_55_KDKRND in LUJV (Figure 4).

The GPC precursor (52.3 kDa, pI = 9.0) is cotranslationally cleaved into the long, stable signal peptide and the mature glycoproteins G1 and G2 [51]–[54]. Based on analogy to LASV [55] and LCMV [56], signalase would be predicted to cleave between D_58_ and S_59_ in LUJV. However, aspartate and arginine residues in the −1 and −3 positions, respectively, violate the (−3,−1)-rule [57]; thus, cleavage may occur between S_59_ and S_60_ as predicted by the SignalP algorithm. The putative 59 aa signal peptide of LUJV displays a conserved G_2_, implicated in myristoylation in JUNV [58], however, it is followed in LUJV by a non-standard valine residue in position +4, resembling non-standard glycine residues found in Oliveros virus (OLVV [59]) and Latino virus (LATV; http://www2.ncid.cdc.gov/arbocat/catalog-listing.asp?VirusID=263&SI=1). Conservation is also observed for aa residues P_12_ (except Amapari virus; AMAV [60]), E_17_ [61](except Pirital virus; PIRV [62]), and N_20_ in hydrophobic domain 1, as well as I_32_KGVFNLYK_40_SG, identified as a CTL epitope in LCMV WE (I_32_KAVYNFATCG; [63]) (Figure 4).

Analogous to other arenaviruses, SKI-1/S1P cleavage C-terminal of RKLM_221_ is predicted to separate mature G1 (162 aa, 18.9 kDa, pI = 6.4) from G2 (233 aa, 26.8 kDa, pI = 9.5) [52],[53],[64]. G2 appears overall well conserved, including the strictly conserved cysteine residues: 6 in the luminal domain, and 3 in the cytoplasmic tail that are included in a conserved zinc finger motif reported in JUNV [65] (Figure 4). G2 contains 6 potential glycosylation sites, including 2 strictly conserved sites, 2 semi-conserved sites N_335_ (absent in LCMVs and Dandenong virus; DANV [19]) and N_352_ (absent in LATV), and 2 unique sites in the predicted cytoplasmic tail (Figure 4). G1 is poorly conserved among arenaviruses [16], and G1 of LUJV is no exception, being highly divergent from the G1 of the other arenaviruses, and shorter than that of other arenaviruses. LUJV G1 contains 6 potential glycosylation sites in positions comparable to other arenaviruses, including a conserved site N_93_HS (Figure 4), which is shifted by one aa in a motif that otherwise aligns well with OW arenaviruses and NW arenavirus clade A and C viruses. There is no discernable homology to other arenavirus G1 sequences that would point to usage of one of the two identified arenavirus receptors; Alpha-dystroglycan (α-DG) [66] that binds OW arenaviruses LASV and LCMV, and NW clade C viruses OLVV and LATV [67], or transferrin receptor 1 (TfR1) that binds pathogenic NW arenaviruses JUNV, MACV, GTOV, and SABV [68] (Figure S2).

In summary, our analysis of the LUJV genome shows a novel virus that is only distantly related to known arenaviruses. Sequence divergence is evident across the whole genome, but is most pronounced in the G1 protein encoded by the S segment, a region implicated in receptor interactions. Reassortment of S and L segments leading to changes in pathogenicity has been described in cultured cells infected with different LCMV strains [69], and between pathogenic LASV and nonpathogenic MOPV [70]. We find no evidence to support reassortment of the LUJV L or S genome segment (Figure 3A and 3B). Recombination of glycoprotein sequence has been recognized in NW arenaviruses [14], [16], [33], [34], [71]–[73], resulting in the division of the complex into four sublineages: lineages A, B, C, and an A/recombinant lineage that forms a branch of lineage A when NP and L sequence is considered (see Figure 3C and 3D), but forms an independent branch in between lineages B and C when glycoprotein sequence is considered (see Figure 3D). While recombination cannot be excluded in case of LUJV, our review of existing databases reveals no candidate donor for the divergent GPC sequence. To our knowledge is LUJV the first hemorrhagic fever-associated arenavirus from Africa identified in the past 3 decades. It is also the first such virus originating south of the equator (Figure 1). The International Committee on the Taxonomy of Viruses (ICTV) defines species within the *Arenavirus* genus based on association with a specific host, geographic distribution, potential to cause human disease, antigenic cross reactivity, and protein sequence similarity to other species. By these criteria, given the novelty of its presence in southern Africa, capacity to cause hemorrhagic fever, and its genetic distinction, LUJV appears to be a new species.

## Materials and Methods

### Sequencing

Clinical specimens were inactivated in TRIzol (liver tissue, 100 mg) or TRIzol LS (serum, 250 µl) reagent (Invitrogen, Carlsbad, CA, USA) prior to removal from BSL-4 containment. Total RNA extracts were treated with DNase I (DNA-free, Ambion, Austin, TX, USA) and cDNA generated by using the Superscript II system (Invitrogen) and 100–500 ng RNA for reverse transcription primed with random octamers that were linked to an arbitrary, defined 17-mer primer sequence [74]. The resulting cDNA was treated with RNase H and then randomly amplified by the polymerase chain reaction (PCR; [75]); applying a 9∶1 mixture of a primer corresponding to the defined 17-mer sequence, and the random octamer-linked 17-mer primer, respectively [74]. Products >70 base pairs (bp) were selected by column purification (MinElute, Qiagen, Hilden, Germany) and ligated to specific linkers for sequencing on the 454 Genome Sequencer FLX (454 Life Sciences, Branford, CT, USA) without fragmentation of the cDNA [19],[76],[77]). Removal of primer sequences, redundancy filtering, and sequence assembly were performed with software programs accessible through the analysis applications at the GreenePortal website (http://156.145.84.111/Tools).

Conventional PCRs at CU were performed with HotStar polymerase (Qiagen) according to manufacturer's protocols on PTC-200 thermocyclers (Bio-Rad, Hercules, CA, USA): an enzyme activation step of 5 min at 95°C was followed by 45 cycles of denaturation at 95°C for 1 min, annealing at 55°C for 1 min, and extension at 72°C for 1 to 3 min depending on the expected amplicon size. A two-step RT-PCR protocol was also followed at CDC using Invitrogen's Thermoscript RT at 60 degrees for 30 min followed by RNase H treatment for 20 min. cDNA was amplified using Phusion enzyme with GC Buffer (Finnzymes, Espoo, Finland) and 3% DMSO with an activation step at 98°C for 30 sec, followed by the cycling conditions of 98°C for 10 sec, 58°C for 20 sec, and 72°C for 1 min for 35 cycles and a 5 min extension at 72°C. Specific primer sequences are available upon request. Amplification products were run on 1% agarose gels, purified (MinElute, Qiagen), and directly sequenced in both directions with ABI PRISM Big Dye Terminator 1.1 Cycle Sequencing kits on ABI PRISM 3700 DNA Analyzers (Perkin-Elmer Applied Biosystems, Foster City, CA).

### Sequence analyses

Programs of the Wisconsin GCG Package (Accelrys, San Diego, CA, USA) were used for sequence assembly and analysis; percent sequence difference was calculated based on Needleman-Wunsch alignments (gap open/extension penalties 15/6.6 for nucleotide and 10/0.1 for aa alignments; EMBOSS [78]), using a Perl script to iterate the process for all versus all comparison. Secondary RNA structure predictions were performed with the web-based version of mfold (http://mfold.bioinfo.rpi.edu); data were exported as .ct files and layout and annotation was done with CLC RNA Workbench (CLC bio, Århus, Denmark). Protein topology and targeting predictions were generated by employing SignalP, and NetNGlyc, TMHMM (http://www.cbs.dtu.dk/services), the web-based version of TopPred (http://mobyle.pasteur.fr/cgi-bin/portal.py?form=toppred), and Phobius (http://phobius.sbc.su.se/). Phylogenetic analyses were performed using MEGA software [79].

## Supporting Information

Figure S1 Phylogenetic tree based on deduced Z amino acid sequence. In contrast to phylogenetic trees obtained with the other ORFs (Figure 2), poor bootstrap support (43% of 1,000 pseudoreplicates) for the branching of LUJV off the LCMV clade was obtained with Z ORF sequence. For GenBank accession numbers see Figure 2.
(0.44 MB TIF)

Figure S2 Pairwise sliding-window distance analysis of GPC sequence. LUJV and members of the OW (LASV, MOPV, IPPYV, LCMV, DANV) and NW (GTOV, CPXV, BNCV, PIRV, OLVV, SABV, MACV) arenavirus complex were compared using LASV NL (A) or GTOV CVH (B) as query (10 aa step; 80 aa window).
(4.21 MB TIF)

Table S1 Pairwise nucleotide and amino acid differences between LUJV and other OW and NW arenaviruses. * NAAV, North American arenavirus. † Values <30% (amino acid) or <33% (nucleotide) are highlighted in green.
(0.20 MB DOC)
