# Supplementary figures and images for: Genetic Detection and Characterization of Lujo Virus, a New Hemorrhagic Fever–Associated Arenavirus from Southern Africa
Source: PLoS Pathog. 2009 May 29;5(5):e1000455. doi: 10.1371/journal.ppat.1000455 (PMC2680969; doi:10.1371/journal.ppat.1000455)

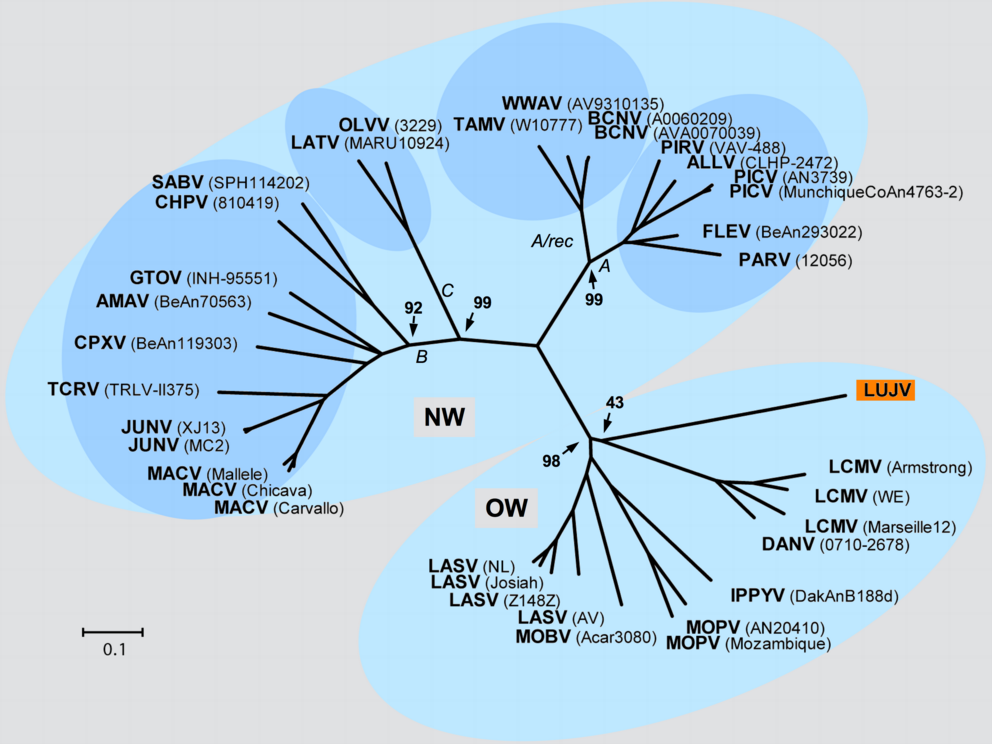

Supplement: Figure S1 — Phylogenetic tree based on deduced Z amino acid sequence. In contrast to phylogenetic trees obtained with the other ORFs (Figure 2), poor bootstrap support (43% of 1,000 pseudoreplicates) for the branching of LUJV off the LCMV clade was obtained with Z ORF sequence. For GenBank accession numbers see Figure 2. (0.44 MB TIF) [file ppat.1000455.s001.tif]
