# Supplementary material for: Genetic Detection and Characterization of Lujo Virus, a New Hemorrhagic Fever–Associated Arenavirus from Southern Africa
Source: PLoS Pathog. 2009 May 29;5(5):e1000455. doi: 10.1371/journal.ppat.1000455 (PMC2680969; doi:10.1371/journal.ppat.1000455)
Supplement: Table S1 — Pairwise nucleotide and amino acid differences between LUJV and other OW and NW arenaviruses. * NAAV, North American arenavirus. † Values <30% (amino acid) or <33% (nucleotide) are highlighted in green. (0.20 MB DOC) [file ppat.1000455.s003.doc]

**Table S1:** Pairwise nucleotide and amino acid differences between LUJV and other OW and NW arenaviruses

|  | **Nucleotide sequence difference (%)** | | | | | | | | | | | | | | | | | | | | | | | | | | | |
| --- | --- | --- | --- | --- | --- | --- | --- | --- | --- | --- | --- | --- | --- | --- | --- | --- | --- | --- | --- | --- | --- | --- | --- | --- | --- | --- | --- | --- |
| Virus | LCMV | DANV | IPPYV | MOPV | MOBV | LASV | LUJV | PARV | PICV | PIRV | ALLV | FLEV | BCNV | CATV | NAAV* | SKTV | WWAV | TAMV | LATV | OLVV | SABV | CHPV | GTOV | AMAV | CPXV | TCRV | JUNV | MACV |
| LCMV | -- | 19.5^†^ | 35.7 | 35.4 | 35.9 | 36.5 | 38.8 | 40.5 | 42.0 | 39.1 | 40.8 | 39.1 | 39.4 | 40.2 | 40.1 | 40.4 | 40.6 | 42.4 | 40.5 | 41.5 | 40.2 | 41.8 | 41.8 | 43.3 | 41.0 | 42.7 | 41.9 | 41.9 |
| DANV | 5.9 | -- | 36.1 | 35.7 | 35.4 | 35.7 | 37.7 | 41.9 | 40.5 | 38.9 | 41.7 | 40.4 | 40.2 | 40.2 | 41.1 | 40.3 | 39.7 | 42.2 | 40.7 | 41.9 | 40.3 | 42.2 | 40.4 | 42.2 | 40.9 | 42.0 | 40.0 | 42.9 |
| IPPYV | 37.2 | 36.6 | -- | 32.9 | 33.9 | 33.4 | 36.3 | 41.8 | 39.9 | 40.6 | 42.2 | 42.2 | 40.9 | 40.3 | 42.6 | 41.3 | 41.2 | 42.3 | 42.0 | 41.4 | 41.4 | 40.5 | 41.8 | 41.8 | 41.3 | 42.1 | 40.9 | 41.0 |
| MOPV | 35.7 | 34.3 | 29.1 | -- | 28.7 | 31.1 | 37.4 | 40.0 | 40.5 | 39.9 | 40.9 | 40.4 | 40.3 | 40.7 | 41.7 | 40.9 | 40.9 | 42.4 | 39.9 | 40.6 | 42.1 | 42.5 | 41.6 | 42.2 | 40.1 | 42.1 | 41.7 | 41.5 |
| MOBV | 38.1 | 36.1 | 29.9 | 21.6 | -- | 31.0 | 37.3 | 39.7 | 40.5 | 40.8 | 39.7 | 40.4 | 41.7 | 39.9 | 39.7 | 41.3 | 41.3 | 41.4 | 41.0 | 41.4 | 41.3 | 41.2 | 41.0 | 42.4 | 40.2 | 41.3 | 42.4 | 41.5 |
| LASV | 39.7 | 38.5 | 31.6 | 26.2 | 27.8 | -- | 38.1 | 41.3 | 39.9 | 41.9 | 40.1 | 40.4 | 40.7 | 40.9 | 40.6 | 41.6 | 41.9 | 40.9 | 39.3 | 41.0 | 41.9 | 43.4 | 40.8 | 43.0 | 41.4 | 41.1 | 41.1 | 41.3 |
| LUJV | 42.1 | 41.8 | 43.8 | 43.9 | 41.2 | 41.2 | -- | 42.2 | 41.9 | 40.7 | 40.4 | 40.4 | 40.9 | 41.1 | 42.6 | 42.6 | 42.8 | 43.4 | 42.3 | 43.0 | 41.3 | 41.9 | 42.5 | 42.3 | 41.9 | 43.3 | 42.6 | 41.7 |
| PARV | 47.1 | 48.0 | 49.6 | 48.4 | 51.3 | 49.7 | 51.0 | -- | 32.4 | 33.9 | 31.7 | 28.7 | 34.9 | 35.2 | 35.7 | 35.6 | 35.4 | 35.6 | 36.6 | 38.3 | 38.7 | 38.6 | 38.7 | 38.5 | 38.0 | 37.9 | 39.4 | 38.1 |
| PICV | 50.2 | 50.8 | 49.8 | 48.5 | 51.7 | 51.7 | 51.5 | 32.7 | -- | 33.4 | 29.5 | 33.3 | 35.1 | 34.3 | 34.7 | 35.7 | 34.3 | 36.5 | 38.4 | 37.1 | 39.1 | 39.3 | 37.8 | 38.6 | 39.1 | 39.5 | 38.5 | 38.7 |
| PIRV | 45.5 | 46.0 | 48.3 | 47.5 | 51.3 | 51.0 | 50.7 | 32.8 | 30.2 | -- | 33.3 | 34.0 | 33.9 | 35.2 | 36.3 | 35.5 | 36.4 | 36.7 | 35.8 | 37.4 | 40.3 | 38.4 | 38.7 | 38.9 | 39.9 | 38.7 | 37.5 | 37.4 |
| ALLV | 48.6 | 48.8 | 50.9 | 48.3 | 51.5 | 48.4 | 52.5 | 28.2 | 23.7 | 30.6 | -- | 30.2 | 35.8 | 34.6 | 33.9 | 33.8 | 35.2 | 35.6 | 37.2 | 36.4 | 38.7 | 39.6 | 39.6 | 38.7 | 37.5 | 38.9 | 37.9 | 37.3 |
| FLEV | 47.5 | 48.5 | 50.8 | 50.6 | 51.5 | 51.5 | 52.5 | 21.4 | 29.9 | 32.6 | 25.5 | -- | 35.7 | 35.0 | 36.1 | 34.9 | 35.3 | 37.0 | 38.1 | 37.6 | 39.2 | 38.9 | 38.3 | 39.6 | 39.0 | 38.7 | 37.6 | 37.6 |
| BCNV | 48.2 | 47.8 | 51.4 | 50.8 | 52.3 | 52.1 | 51.0 | 34.3 | 35.9 | 36.4 | 35.6 | 34.8 | -- | 27.9 | 27.4 | 26.1 | 27.6 | 28.0 | 38.3 | 38.1 | 37.1 | 38.9 | 38.4 | 39.0 | 39.2 | 38.2 | 39.7 | 37.8 |
| CATV | 50.8 | 51.2 | 53.3 | 50.0 | 51.1 | 51.1 | 53.8 | 38.2 | 36.2 | 38.2 | 38.4 | 35.6 | 17.8 | -- | 21.3 | 22.5 | 22.8 | 26.7 | 38.1 | 39.0 | 37.7 | 37.2 | 38.2 | 39.4 | 38.1 | 39.6 | 39.4 | 38.1 |
| NAAV* | 51.1 | 50.0 | 52.1 | 48.3 | 50.8 | 52.0 | 53.0 | 37.7 | 33.9 | 38.3 | 36.8 | 36.7 | 19.6 | 13.2 | -- | 24.7 | 19.2 | 27.4 | 38.5 | 38.3 | 37.5 | 37.8 | 37.9 | 39.5 | 39.1 | 39.0 | 39.6 | 39.2 |
| SKTV | 49.4 | 50.3 | 52.3 | 48.8 | 51.3 | 51.5 | 53.0 | 38.4 | 36.2 | 37.9 | 37.2 | 37.0 | 16.9 | 11.4 | 15.5 | -- | 22.6 | 26.7 | 38.1 | 39.6 | 39.6 | 38.3 | 37.1 | 39.4 | 39.8 | 39.6 | 39.6 | 39.6 |
| WWAV | 50.2 | 50.7 | 52.1 | 48.1 | 50.3 | 51.8 | 53.2 | 39.0 | 35.0 | 37.4 | 36.6 | 37.5 | 18.3 | 13.5 | 9.1 | 14.2 | -- | 28.2 | 38.3 | 38.5 | 37.6 | 39.5 | 38.5 | 39.5 | 38.8 | 39.3 | 40.0 | 39.0 |
| TAMV | 50.8 | 51.1 | 55.0 | 51.2 | 53.3 | 54.4 | 55.0 | 37.7 | 36.5 | 38.4 | 40.2 | 38.3 | 21.4 | 18.5 | 19.4 | 19.0 | 19.9 | -- | 37.5 | 39.1 | 38.9 | 40.6 | 38.3 | 41.0 | 40.5 | 38.8 | 40.0 | 39.7 |
| LATV | 48.5 | 48.2 | 50.8 | 48.3 | 49.7 | 50.3 | 52.1 | 41.5 | 42.5 | 41.0 | 40.8 | 43.1 | 41.4 | 43.4 | 41.9 | 41.9 | 41.6 | 43.0 | -- | 27.3 | 37.3 | 36.8 | 35.1 | 36.2 | 35.8 | 36.6 | 36.3 | 35.3 |
| OLVV | 49.8 | 48.8 | 51.1 | 49.9 | 49.6 | 49.2 | 53.2 | 42.7 | 43.3 | 41.8 | 41.8 | 41.9 | 43.4 | 44.4 | 43.4 | 45.3 | 43.9 | 42.6 | 19.2 | -- | 38.9 | 37.2 | 36.9 | 35.5 | 35.8 | 37.6 | 34.7 | 35.2 |
| SABV | 48.4 | 49.4 | 49.8 | 51.0 | 49.8 | 49.7 | 51.0 | 43.9 | 44.6 | 44.2 | 44.0 | 43.1 | 44.5 | 45.9 | 44.2 | 45.5 | 45.0 | 45.2 | 38.5 | 38.6 | -- | 24.8 | 32.8 | 32.1 | 32.6 | 34.2 | 32.0 | 31.7 |
| CHPV | 48.5 | 48.7 | 49.7 | 48.8 | 50.7 | 49.7 | 51.7 | 40.9 | 44.3 | 42.4 | 42.6 | 40.7 | 44.8 | 45.7 | 43.7 | 44.6 | 45.9 | 45.1 | 38.6 | 38.4 | 16.2 | -- | 31.5 | 30.6 | 32.4 | 32.9 | 31.9 | 33.3 |
| GTOV | 48.8 | 50.0 | 49.7 | 48.9 | 49.6 | 49.5 | 52.7 | 42.1 | 43.3 | 42.5 | 43.3 | 41.4 | 45.1 | 45.2 | 44.3 | 45.5 | 44.6 | 47.3 | 35.2 | 37.7 | 29.5 | 27.8 | -- | 24.5 | 26.0 | 31.8 | 30.1 | 30.3 |
| AMAV | 48.5 | 49.2 | 49.0 | 48.4 | 49.7 | 49.3 | 52.9 | 43.0 | 43.0 | 43.6 | 42.2 | 42.1 | 45.0 | 46.4 | 44.8 | 46.9 | 45.2 | 47.4 | 37.2 | 37.7 | 27.8 | 27.3 | 14.6 | -- | 26.2 | 32.0 | 31.1 | 31.4 |
| CPXV | 47.1 | 48.4 | 49.8 | 48.2 | 48.6 | 48.8 | 51.2 | 44.2 | 45.4 | 45.0 | 43.0 | 44.0 | 47.1 | 46.8 | 46.2 | 48.0 | 46.4 | 47.0 | 35.5 | 36.1 | 29.3 | 28.7 | 17.1 | 17.1 | -- | 32.1 | 31.1 | 30.1 |
| TCRV | 49.3 | 49.3 | 50.3 | 50.3 | 51.0 | 50.2 | 52.4 | 43.8 | 45.1 | 44.2 | 42.7 | 44.5 | 45.6 | 47.0 | 44.6 | 46.7 | 45.8 | 47.6 | 40.2 | 40.7 | 34.3 | 32.2 | 29.5 | 30.5 | 30.9 | -- | 27.1 | 27.3 |
| JUNV | 50.4 | 50.8 | 51.1 | 51.0 | 51.3 | 51.1 | 53.2 | 43.0 | 44.5 | 43.8 | 42.6 | 41.5 | 46.8 | 46.0 | 44.2 | 47.2 | 45.3 | 46.1 | 39.2 | 39.7 | 30.3 | 30.7 | 25.9 | 26.8 | 27.7 | 22.1 | -- | 23.8 |
| MACV | 49.1 | 50.4 | 50.5 | 50.8 | 50.3 | 50.0 | 52.7 | 43.7 | 45.0 | 44.2 | 42.0 | 42.4 | 47.2 | 45.8 | 45.2 | 47.6 | 45.2 | 47.0 | 37.4 | 38.5 | 28.7 | 29.4 | 24.3 | 26.2 | 26.1 | 21.2 | 12.2 | -- |
|  | **Amino acid sequence difference (%)** | | | | | | | | | | | | | | | | | | | | | | | | | | | |

* NAAV – North American arenavirus. † Values <30% (amino acid) or <33% (nucleotide) are highlighted in green.
